# Thin‐Plate Superstructures of the Immunogenic 33‐mer Gliadin Peptide

**DOI:** 10.1002/cbic.202200552

**Published:** 2022-10-18

**Authors:** Maria Georgina Herrera, Maria Julia Amundarain, Franscesco Nicoletti, Marcus Drechsler, Marcelo Costabel, Pier Luigi Gentili, Veronica Isabel Dodero

**Affiliations:** ^1^ Faculty of Chemistry OCIII Bielefeld University Universitätsstr. 25 33615 Bielefeld Germany; ^2^ Faculty of Exact and Natural Sciences Institute of Biosciences Biotechnology and Translational Biology (iB3) University of Buenos Aires Intendente Güiraldes 2160, Ciudad Universitaria C1428EGA Buenos Aires Argentina; ^3^ Instituto de Física del Sur (IFISUR) Departamento de Física Universidad Nacional del Sur (UNS) CONICET Av. L. N. Alem 1253, B8000CPB - Bahía Blanca Argentina; ^4^ Bavarian Polymer Institute University Bayreuth Universitaetsstr. 30 95447 Bayreuth Germany; ^5^ Department of Chemistry, Biology, and Biotechnology Università degli Studi di Perugia Via Elce di Sotto 8 06123 Perugia Italy

**Keywords:** 33-mer gliadin peptides, PPII secondary structure, self-assembly, supramolecular chemistry, thin-plate structures

## Abstract

Gluten related‐disorders have a prevalence of 1–5 % worldwide triggered by the ingestion of gluten proteins in wheat, rye, barley, and some oats. In wheat gluten, the most studied protein is gliadin, whose immunodominant 33‐mer amino acid fragment remains after digestive proteolysis and accumulates in the gut mucosa. Here, we report the formation of 33‐mer thin‐plate superstructures using intrinsic tyrosine (Tyr) steady‐state fluorescence anisotropy and cryo‐TEM in combination with water tension measurements. Furthermore, we showed that fluorescence decay measurements of 33‐mer intrinsic fluorophore Tyr provided information on the early stages of the formation of the thin‐plate structures. Finally, conformational analysis of Tyr residues using minimalist models by molecular dynamic simulations (MD) demonstrated that changes in Tyr rotamer states depend on the oligomerization stage. Our findings further advance the understanding of the formation of the 33‐mer gliadin peptide superstructures and their relation to health and disease.

## Graphical abstract

**The immunogenic 33‐mer** gliadin peptide builds up thin‐plate superstructures that could be responsible for an innate immune response in the context of gluten‐related disorders. A multi‐technique evaluation provides insight into the self‐assembly process showing that the intrinsic tyrosine fluorescence is a sensor for monitoring the formation of the thin‐plate structures.

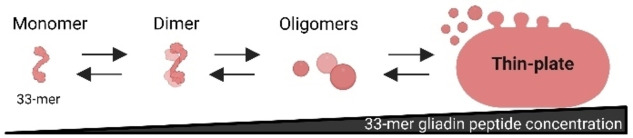

## Introduction

Gluten‐related disorders are a group of diseases that involve the activation of the immune system triggered by the ingestion of gluten, leading to celiac disease^[1]^ (CeD), wheat allergy, and gluten or wheat non‐celiac sensibility. These disorders have a high prevalence in Western societies, around 1–5 % worldwide because gluten is present in wheat, rye, barley, and some varieties of oats.^[2]^ The peptides obtained after the incomplete proteolysis of the gluten proteins are responsible for CeD in susceptible individuals, but their role remains elusive in other gluten‐related disorders.^[3]^ Recently, it was shown that during pepsin proteolysis of the wheat gluten protein gliadin, the resulting proteolytical‐resistant peptides form amyloid‐like aggregates spontaneously. Importantly, gliadin peptide aggregates triggered pro‐inflammatory and pro‐apoptotic markers in cellular models.^[4]^ This report demonstrated the capability of gliadin aggregates as novel structural features in connection with pathogenicity. Among the different gliadin fragments, it is accepted that a 33‐mer amino acid fragment of gliadin remains intact after proteolysis by the enzymes of the human gut brush border and in perfusion experiments with rats.^[5]^ In this regard, it is accepted that the 33‐mer is the immunodominant peptide related to CeD, and their accumulation could be a trigger of CeD.^[6]^ At the molecular level, the 33‐mer gliadin peptide, ^57^LQLQPFPQ(PQPQLPY)_3_PQPQPF^89^ contains 33 % of proline (11 residues), leading to a type‐II polyproline (PPII) conformation which is known to be bound to MHC class‐II molecules in CeD patients.^[6a]^ The 33‐mer also has 24 % of glutamine (8 residues) that confers an amphipathic nature, 15 % of aromatic amino acids, tyrosine (Tyr, 3 residues), phenylalanine (2 residues), and 17 % of leucine (5 residues).

In the last years, it was demonstrated that the 33‐mer forms dynamic small oligomers and large aggregates depending only on peptide concentration with a concomitant conformational transition from polyproline II (PPII) to the parallel beta structure under aqueous conditions.^[7]^ Previously, enzymatic Tyr‐Tyr crosslinking was employed to stabilize the 33‐mer peptide small oligomers, from dimers to decamers. It was also shown by all‐atom molecular dynamics simulations (MD) that the complementary partial charge distribution and its left‐handed helical structure PPII are responsible for the self‐assembly.^[8]^ Although there is an increment in the content of the beta structure on increasing peptide concentration,^[7a]^ these highly dynamic oligomers do not bind to Thioflavin T (ThT), a dye used to detect amyloid‐type aggregates, limiting ThT use to detect the formation of the large 33‐mer aggregates in real‐time.^[9]^ While the primary structure of 33‐mer is associated with the adaptive immune response in CeD patients, it was found that the large aggregates trigger an innate immune response *in vitro* experiments with macrophages mediated by Toll‐Like Receptors 4 and 2 (TLR2 and 4).^[10]^

It is accepted that the amount of gluten intake matters for triggering CeD.^[11]^ The average gluten intake in the general population is around 10–20 grams of gluten daily.^[12]^ The same amount has been shown to be sufficient to establish a dose‐dependent response to enteropathy in CeD patients after 4 to 8 weeks of gluten challenge.^[13]^ Considering the relevance of the proteolysis‐resistant 33‐mer in CeD, its concentration in wheat flour was quantified as 90.9–602.6 μg per gram of flour depending on wheat cultivars.^[14]^ If it is considered that the average gluten in wheat flour is 10 %,^[15]^ that means for 10 g of gluten, a daily intake of 0.91 mg—6.0 mg of 33‐mer is possible. Although human nutrition is highly complicated and different factors, like microbiota and individual characteristics are important, it seems to be possible that the local concentrations of the 33‐mer *in vivo* would be high enough for the formation of 33‐mer nanostructures detected *in vitro* in the concentration range from 60–600 μM (0.24—2.4 mg/mL). Therefore, the accumulation and formation of the large 33‐mer aggregates with the activation of immune cells could be an unrecognized trigger event in CeD that requires further biomedical investigations.

In the meantime, the characterization of the 33‐mer small oligomers was straightforward because the system reaches a metastable equilibrium that depends on peptide concentration.[^7^, ^10^] Therefore, different microscopic methods, including atomic force microscopy (AFM), transmission electron microscopy (TEM), scanning electron microscopy (SEM), and helium ion microscopy (HIM), demonstrated that the small 33‐mer oligomers have a size of 20–30 nm with a spherical and rod‐like shape. Although dynamic light scattering (DLS) experiments showed the co‐existence of small and large 33‐mer aggregates, the morphology of the large 33‐mer structures in solution remains elusive mainly because of the substantial increment of the peptide concentration[^7a^, ^10^] during the deposition process on surfaces or under vacuum, limiting the information provided by AFM or TEM.[^7^, ^10^]

Here, we envisaged a multi‐technique investigation to elucidate the formation and morphology of the large 33‐mer supramolecular structures. First, we investigated the process of the 33‐mer gliadin peptide self‐assembly in the micromolar range (100–600 μM) by intrinsic Tyr steady‐state anisotropy, complementing it with surface tension measurements below 100 μM and cryo‐TEM at 600 μM. Second, we monitored the formation of the 33‐mer large superstructures by employing the Tyr fluorescence decay measurements at different peptide concentrations to determine the time required for the excited state of Tyr residues to decay 1/e of the total population which is known as the lifetime of the excited state.^[16]^ We implemented two types of analysis: the exponential model through the non‐linear least squares (NLLS) methodology, which adjust the decay to exponential curves to obtain the lifetimes’ values,^[17]^ and the maximum entropy method (MEM), which recovers the fluorescence decay times without limiting the number of exponential terms needed to fit the fluorescence decays.^[18]^ Finally, we present a conformational analysis of Tyr residues in the 33‐mer peptide in a dimer and a decamer as minimalist models for the self‐assembly model by MD simulations. Our report contributes to understanding the formation of 33‐mer superstructures with the final aim to elucidate their role in health and disease.

## Results and Discussion

### The self‐assembly of 33‐mer gliadin peptide starts in the nanomolar concentration range forming thin plates at high concentrations

**Figure 1:**
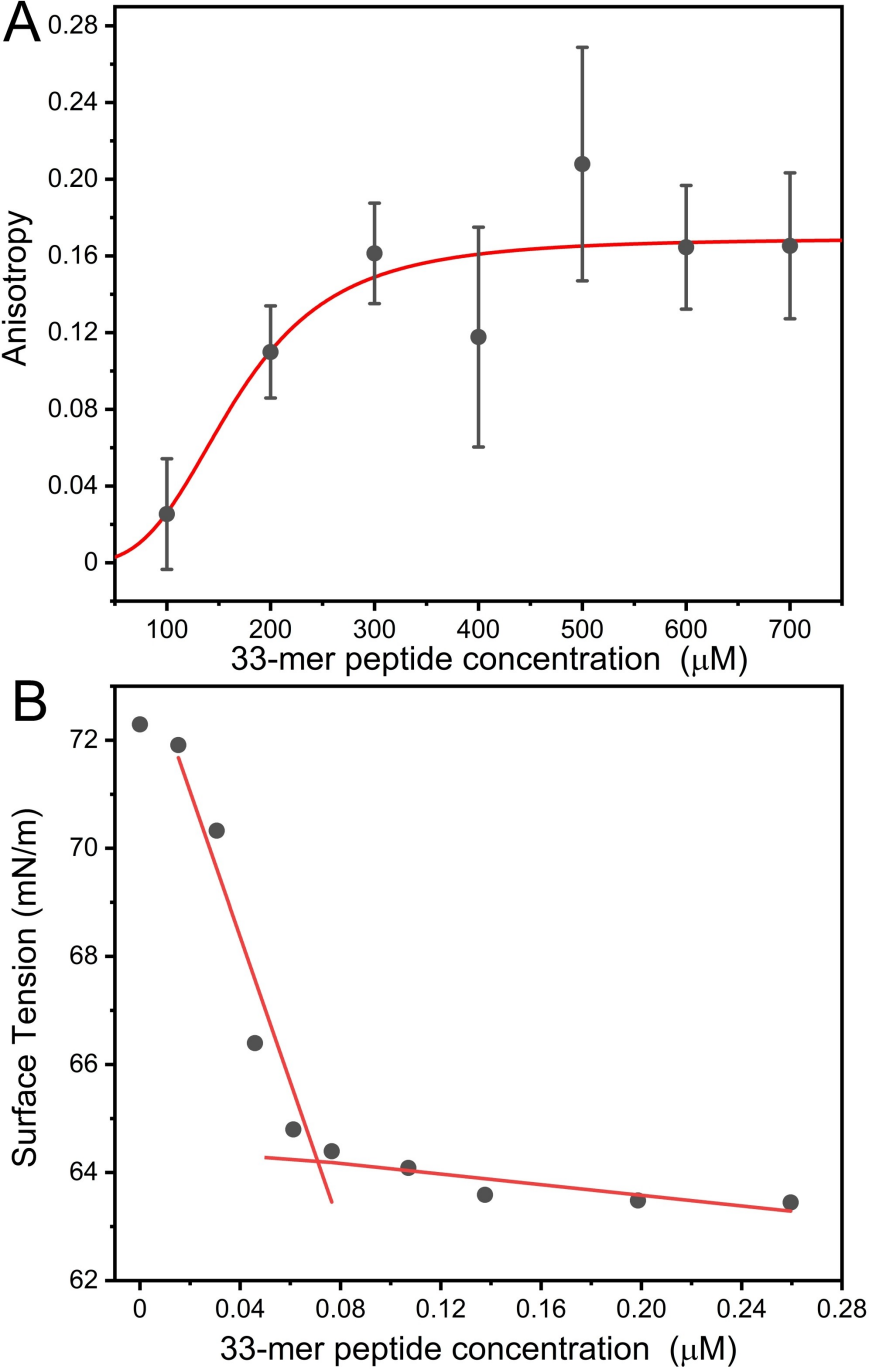
Analysis of the self‐assembly of the 33‐mer gliadin peptide. A) Anisotropy of Tyr residues at different concentrations in the micromolar range. B) Water surface tension measurements at increasing 33‐mer concentrations in the nanomolar range obtained by the Wilhelmy method at 22 °C.

Firstly, we measured the steady‐state intrinsic Tyr fluorescence anisotropy of a stabilized 33‐mer aqueous solution at pH 6.5 by increasing the peptide concentration. Below 100 μM, the anisotropy signal was negligible. Similarly, it was reported that below 125 μM, there was no detectable DLS signal.^[7a]^ In Figure 1A, it is shown the trend of Tyr anisotropy as a function of 33‐mer peptide concentration. Tyr anisotropy increases from 0.028±0.02 up to 0.16±0.038 when 33‐mer concentration increases from 100 μM up to 300 μM. It reaches a plateau at 0.16 within the experimental error.

This trend of anisotropy is probably due to a gain of order around the phenol group of the Tyr residue promoted by a self‐assembly process. This agrees with previous observations by TEM and DLS, where the presence of oligomers and large structures within this concentration range was detected. Fitting this result with a simple one‐to‐one binding model and considering the association of oligomers into the larger structures, we obtained a dissociation constant (KD) of 165 μM. Previously, it was detected that a pronounced change in the secondary structure from PPII towards β‐sheet occurs around 200 μM by circular dichroism (CD) and ATR‐FTIR.^[7]^ Together, these results confirm the formation of larger 33‐mer superstructures above 165 μM.

To investigate the minimum concentration at which the peptide oligomerizes, we hypothesized that spectroscopic techniques could not be suitable to detect the primordial oligomerization stages of 33‐mer as dimer or trimer because of the low signal/noise ratio below the micromolar range. Therefore, based on the proposed amphipathic character of 33‐mer,^[7]^ we measured the water surface tension by the Wilhelmy method, as it was previously done for gliadin^[4]^ and zein peptides.^[19]^

We measured freshly filtered 33‐mer peptide, which decreased the water surface tension from 72.1 mN/m to 63.8 mN/m, obtaining a critical concentration of aggregation (CAC) as low as 75 nM (Figure 1B). Subsequent additions of the peptide to the subphase slightly modified the obtained value, thus suggesting that the system reaches equilibrium at the air‐liquid interface. This result implies that the 33‐mer adsorbs at the air‐water interface, as other surfactant‐like peptides do. Above the CAC, the 33‐mer monomer might enter the subphase and start forming early micellar‐like aggregates. The CAC obtained was much lower than the detection limit of the previously used methods, namely, DLS, spectroscopy, and microscopy. We hypothesized that above the CAC, the 33‐mer could form small oligomers, ranging from dimers to decamers, as found previously by 33‐mer crosslinking experiments.^[12]^ Previously, Kogan *et al*. showed similar behavior of proline and glutamine‐rich domain from zein protein.^[20]^ They showed that the surfactant activity gives the property to penetrate cells. We hypothesized that the amphiphilic nature of the 33‐mer could explain the now‐controversial transport mechanism of the 33‐mer;^[21]^ however, it requires further investigations that exceed this report.

**Figure 2:**
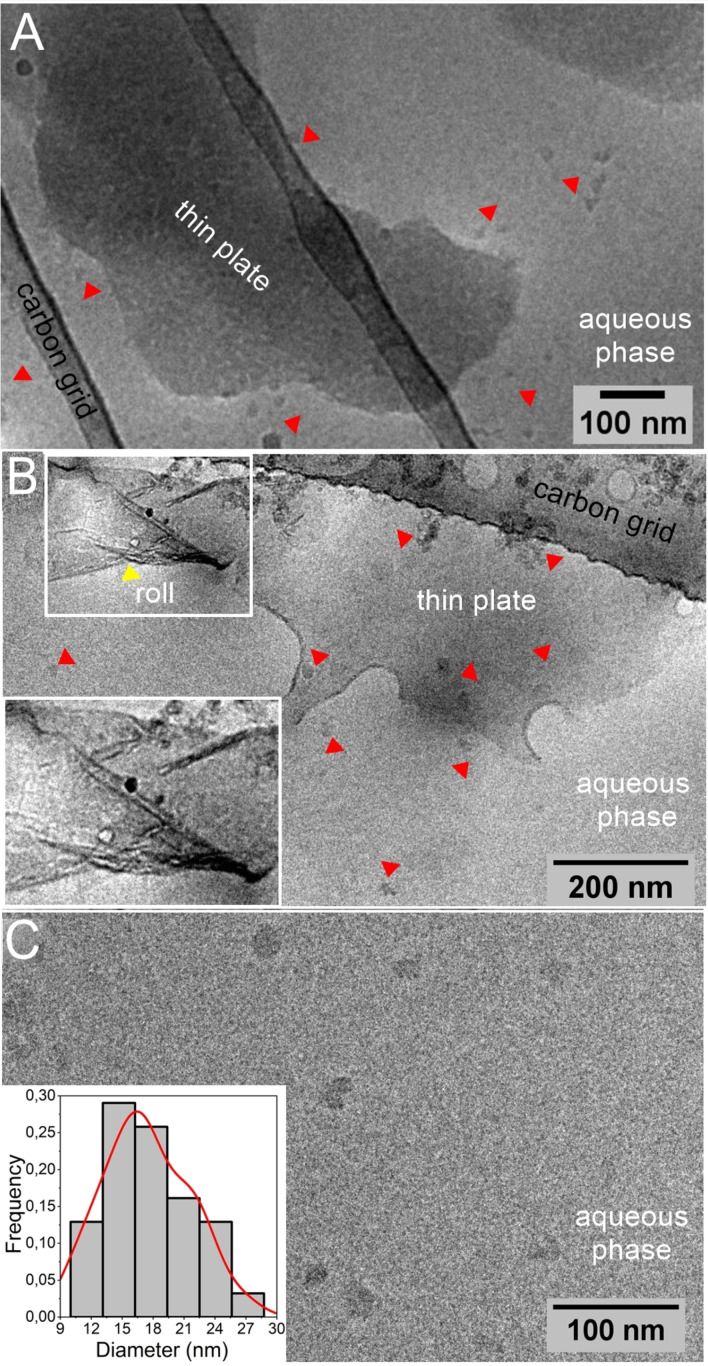
The 33‐mer gliadin peptide is a non‐ionic peptide amphiphile self‐assembling into thin‐plate superstructures on increasing peptide concentration in the micromolar range. A) Cryo‐TEM images of 33‐mer thin‐plate or sheet‐like structures and oligomers (red arrowheads) at 600 μM in water MilliQ. B) After being vortexed the specimen, the thin‐plates structures remain, and it is possible to visualize how they roll up (inset). C) Magnified image of 33‐mer oligomers; the insert presents the statistical distribution of 33‐mer gliadin oligomers (N=31, 17±4 nm).

Next, we investigated the morphology of 33‐mer larger superstructures near a native state by cryo‐TEM. Previously, cryo‐TEM was successfully used to characterize other protein self‐assembled systems, such as alpha‐synuclein oligomers^[22]^ and amyloid peptide channel‐like protofibrils.^[23]^ By the thin‐film freezing method,^[24]^ we detected the presence of large 2D thin‐plate structures and oligomers of 17±4 nm at 600 μM (Figure 2 panels A–B, in arrowheads, are shown the oligomers). In the whole observation field, we observed the co‐existence of the large superstructures with oligomers and some oligomers agglomerates (Figure S2). By being vortexed the 33‐mer self‐assembled system, the thin sheet rolled, folded, and remained stable (Figure 2 panel B). The fact that the dispersion does not precipitate might be due to the presence of stabilized planar structures,^[25]^ as observed previously in other peptide systems, such as Phe‐Phe or protein detergents.^[26]^ Namm *et al*. reported the formation of free‐floating ultrathin 2D sheets from self‐assembling peptides.^[27]^ Hamley *et al*. reported the occurrence of floating nanosheets as precursors of filaments and nanotubes in an amphiphilic arginine‐coated peptide.^[28]^

Different structures in the 33‐mer dispersion correlate quite well with the previous DLS experiments that small oligomers coexist with large superstructures.[^7a^, ^10^] Moreover, the simultaneous formation of planar structures stabilized in solution and the visualization of small oligomers support previous AFM and TEM observations of dehydrated plaques or ordered linear arrays surrounded by oligomers under deposition conditions (Figure S3).[^7a^, ^10^] In other amyloid systems, like α‐synuclein, it is frequently observed the presence of more than one nanostructured specie, e. g., transient oligomers with different morphology, as well as the co‐existence of fibrils and oligomers. Interestingly, there are an increased number of studies showing that the initially formed oligomers that build up the fibrils are different from those isolated when the fibrils are formed which is excellent reviewed recently by Bigi *et al*.^[29]^ In this context, Cascella *et al*. found that those release oligomers from α‐synuclein fibrils are the toxic species inducing dysfunction in neuronal cells.^[30]^

In the case of 33‐mer, the thin plates do not show high order. We hypothesize that the interaction between the 33‐mer oligomers led to the formation of the observed thin 2D structures with increasing peptide concentration, similar to other cell‐penetrating peptides with PPII structures,[^19^, ^20^] however it could be also the case that they are new oligomers that are released from the thin‐plate structure.

### Fluorescence decay measurements of the excited state of 33‐mer intrinsic fluorophore Tyr monitor aggregation changes from oligomers to thin‐plate structures

Considering the results obtained by anisotropy and the observation of the thin‐plate structures, we hypothesized that fluorescence decay measurements of the Tyr residues could provide information about the self‐assembly process of the 33‐mer gliadin peptide, as it was shown for the alpha‐synuclein protein.^[31]^ In general, the multiexponential decay observed in intrinsic protein fluorescence could be due to micro‐heterogeneity at the intra‐ and inter‐entities levels. Micro‐heterogeneity is at the intra‐entity level due to multiple ground‐state conformations, named rotamers,^[32]^ and dielectric relaxations of the excited states.^[33]^ It is at the inter‐entity level due to distinct aggregates and micro‐environments.^[34]^ In recent works, the study on the rotamer distribution of Tyr residues was used to evaluate how their changes correlate with other system properties, such as secondary structure and aggregation propensity.^[35]^ Considering that the three Tyr residues of 33‐mer are located in the repetitive region PQLPYPQPQ, we simplified the analysis by assuming that they have similar photophysical behavior and that any change is related to a self‐assembly process.

**Figure 3:**
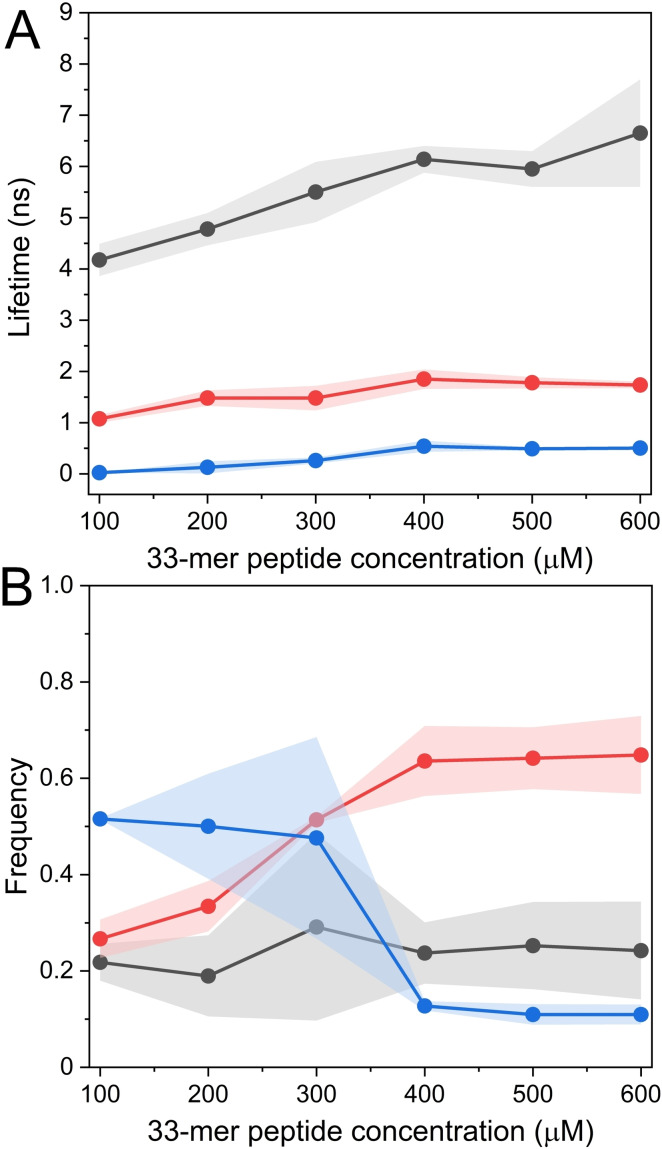
Time correlated photon counting of the 33‐mer gliadin peptide in water from 100 to 600 μM was analyzed with the non‐linear least‐squares method. A) Tyr fluorescence decay times τ1 (black), τ2 (red), and τ3 (blue) were obtained for each concentration B) Values of the fraction of intensity obtained from the three lifetimes previously described f1 (black), f2 (red), and f3 (blue).

Next, we applied time‐correlated photon counting spectroscopy in the concentration range from 100 to 600 μM. As we previously reported, the 33‐mer colloidal system reaches equilibrium after three days at 4 °C, where the large superstructures are detected at 600 μM. The 33‐mer superstructures are stable, as shown here by Cryo‐TEM. However, they undergo a reversible disaggregation by filtration or dilution.[^7b^, ^10^] Only equilibrated samples are reported to simplify the analysis. After the data acquisition and fitting by a non‐linear least squares method, we obtained that three possible lifetimes contribute to the overall decay, named τ1, τ2, and τ3. Each lifetime contributes differently, as presented in Figure 3 and Table 1. It was detected that there is an increment in the Tyr lifetimes over the concentrations tested. This effect was pronounced in τ1, whose values increased from 4.80 ns to 6.65 ns. The analysis of the contribution of each lifetime to the overall decay shows that the f1 stays constant primarily along the whole concentration range; however, f3 and f2 are the ones that change mainly over the analyzed range. Interestingly, both f3 and f2 contributions decrease after 100 μM, and both reach a plateau at around 400 μM (Figure 3B). In this sense, we propose that the changes in the lifetimes and their contributions correlate with the shift in tyrosine rotamer species during the formation of the thin‐plate structures. Similar results were observed in the β‐amyloid peptide, where the formation of fibrils at high concentration was translated into a change in the contribution of each lifetime due to the aggregation process.^[36]^

**Table 1:** Comparison between the lifetimes obtained by NLLS and MEM methods. Also, we present for each lifetime (τ) the frequency contribution (*f*) for NLLS and the weight in percentage (*w* ( %)) obtained by MEM. In addition, it presents the values of fuzzy entropy (H).

33‐mer	τ1				τ2				τ3				Fuzzy
(μM)	NLLS		MEM		NLLS		MEM		NLLS		MEM		entropy (H)
	τ (ns)	*f1*	τ (ns)	*w* (%)	τ (ns)	*f2*	τ (ns)	*w* (%)	τ [ns)	*f3*	τ (ns)	*w* (%)	
100	4.80	0.21	3.38	0.54	1.08	0.27	0.90	3.53	0.02	0.52	0.02	0.11	0.79
200	4.48	0.19	6.06	0.96	1.48	0.33	2.19	6.70	0.13	0.50	1.50	1.83	0.69
300	5.50	0.29	7.07	0.81	1.48	0.51	2.12	9.24	0.26	0.47	1.05	1.15	0.73
400	6.13	0.24	7.76	0.58	1.85	0.64	2.06	11.40	0.54	0.13	1.50	1.37	0.66
500	5.95	0.25	6.25	1.15	1.78	0.64	1.94	15.00	0.49	0.11	1.61	3.54	0.56
600	6.65	0.24	6.25	1.14	1.85	0.65	1.94	14.05	0.51	0.11	1.50	1.17	0.66

### The maximum entropy method (MEM) analysis confirms the presence of different tyrosine populations

**Figure 4:**
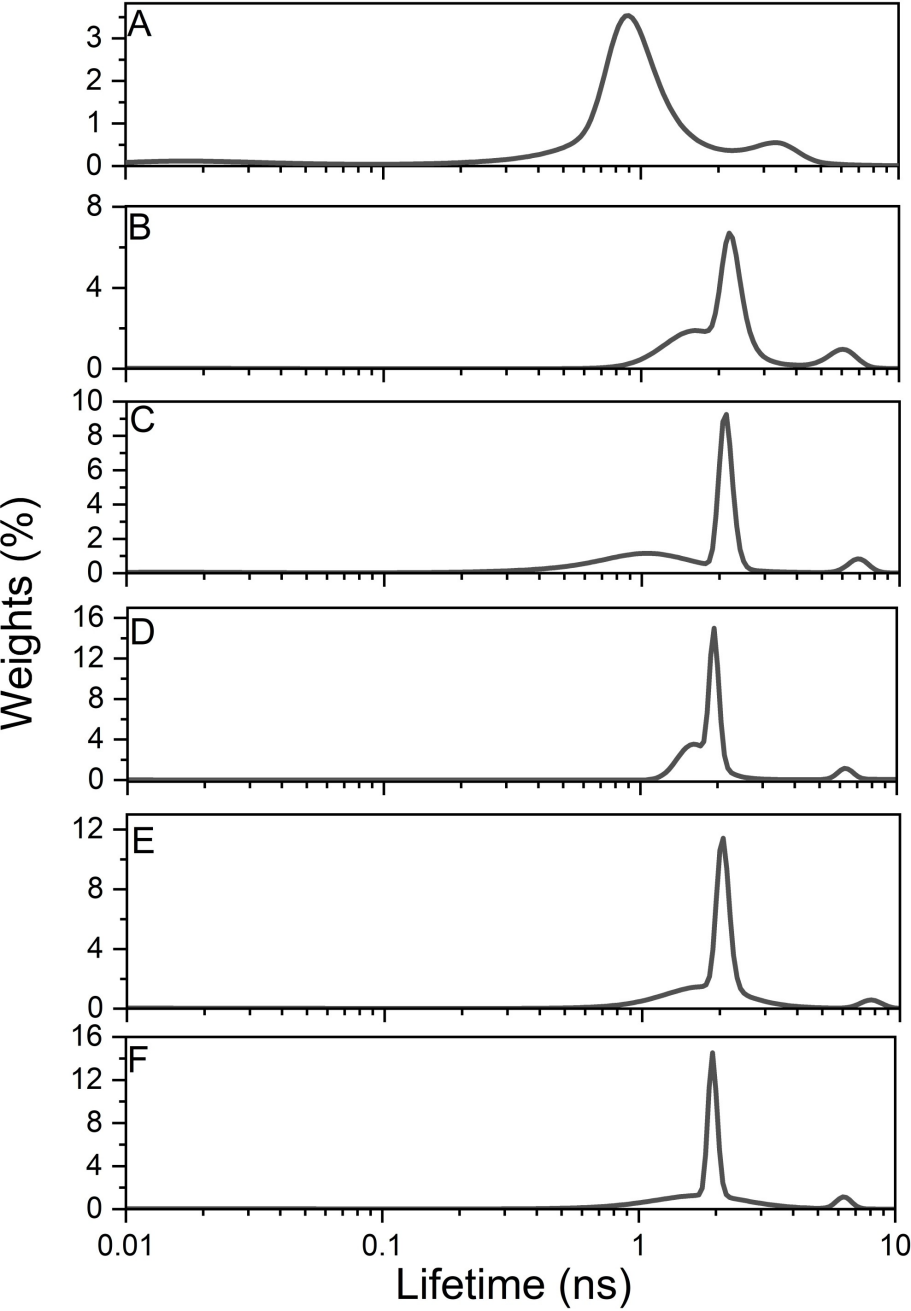
MEM analysis of the 33‐mer gliadin peptide Tyr fluorescence decays depending on peptide concentration. A) 100 μM, B) 200 μM, C) 300 μM, D) 400 μM, E) 500 μM and F) 600 μM.

The analysis of the fluorescence decays kinetics by MEM allows more insight into the self‐assembly events with the 33‐mer gliadin peptide because, as mentioned before, it is not limiting the number of exponential terms needed to fit the fluorescence decays. At 100 μM, three peaks correspond to three Tyr fluorescence decay lifetimes of the excited state (Figure 4A); the principal one is a band that peaked at 0.90 ns (τ2, with a percentage weight of 3.53) and included between 0.5 and 1.9 ns (Table 1); the second band in decrease order of intensity is centered at 3.38 ns (τ1, having a percentage weight of 0.54) and is included between 1.9 and 5 ns. The third one peaked at 0.017 with a minor contribution (τ3, percentage weight of 0.11). The degree of micro‐heterogeneity can be quantitatively estimated by determining its Fuzzy Entropy (H), which indicates a higher degree of flexibility in the system when its value is near 1 (the Experimental Section reports its definition).[^35^, ^37^] At 100 μM, H was 0.79.

An increase in the peptide concentration from 100 to 200 μM significantly affects the lifetimes’ distribution (Figure 4B). There are still three bands related to the Tyr lifetimes; However, the principal band is shifted from 0.9 to 2.19 ns (τ2, with a percentage value of 6.7) (Table 1) and included between 1.8 and 3.0 ns. The second band is also shifted from 3.38 to 6.06 ns (τ1, with a weighted percentage of 0.96), where the 6.06 ns band is included between 4 and 8 ns. The third one appears as a shoulder, which is centered at 1.5 ns (τ3, with a weighted percentage of 1.83), and it was shifted from 0.017 ns. The Fuzzy Entropy slightly decreases to 0.69. These results highlight that the formation of the thin planar structures has a concrete repercussion on the lifetimes’ distribution for the Tyr residues.

An increase in peptide concentration from 200 to 300 μM (Figure 4C) does not change the values of Tyr fluorescence time decays significantly, but the weighted percentage of only τ2 increases considerable: the principal band is centered at 2.12 ns (τ2, with a weighted percentage of 9.24) and is included between 1.8 and 2.5 ns; the second one (τ1) is centered at 7.07 ns and is comprised between 5.5 and 8.8 ns. The third one (τ3) is centered at 1.05 ns and has a percentage weight of 1.15. Beyond 300 μM, the Tyr fluorescence decay times through MEM values are very similar, with an average H of 0.66 (Table1). The dominant band of τ2 peaked at around 2 ns, with a maximum weight larger than 11. The second band is beyond 6 ns and has a much small weight (between 0.58 and 1.15 %). The third one is around 1.5 ns with an average weight of 2. Their Fuzzy Entropies slightly decrease up to 0.66 (Table1), which indicates that the thin plates formed are stabilized, as evidenced by cryo‐TEM (see Figure 2) and the trend of fluorescence anisotropy (see Figure 1A).

Since the significant change in the Tyr fluorescence decay times was from 100 to 200 μM, this validated the KD of 165 μM obtained by the Tyr anisotropy experiments. Additionally, we hypothesized that the most dynamic band corresponding to τ3 could be a signature of the smaller oligomers.

Altogether these results show that analyzing the changes in tyrosine lifetime is a valuable method to follow the self‐assembly of 33‐mer gliadin peptide into larger structures.

### Tyrosine rotamers in 33‐mer change during decamer formation by molecular dynamics simulations

**Figure 5:**
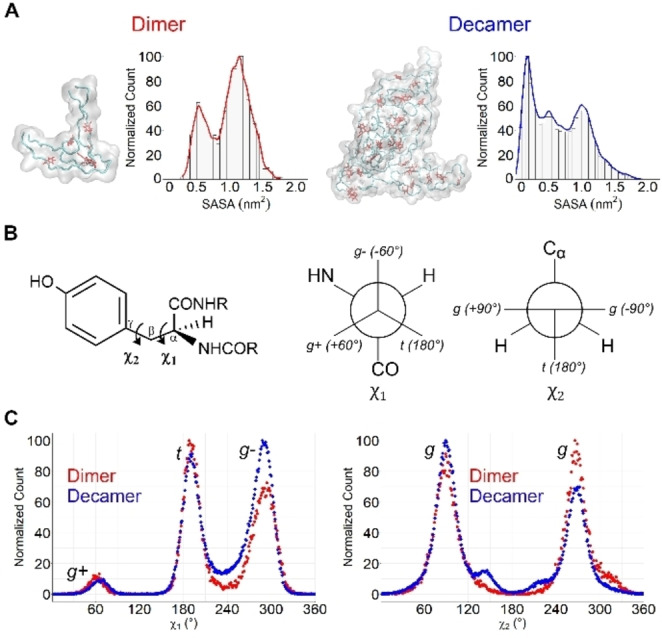
MD Simulations show that different oligomeric species prefer certain rotamer states. A) Snapshot of the stabilized oligomers dimer and decamer and their respective normalized histogram of the Tyr solvent accessible surface area in the last 100 ns of their trajectories. B) Tyrosine residue with the definition of the χ_1_ and χ_2_ angles and the rotamers definition. C) Plots of the χand χdistribution in the last 100 ns of the trajectories of the dimer (red) and decamer (blue). Each point represents the normalized total number of occurrences. The axis values in χ_1_ are in the range of 0–360° for clarity.

Finally, we analyzed two MD simulations^[8]^ performed on a pair of monomers and ten monomers randomly distributed in water, producing a stable dimer and a decamer, respectively (Figure 5). We calculated the solvent accessible surface area (SASA) of the lateral chains of the Tyr residues to assess the overall location of the tyrosine residue in the oligomers, namely: in the core and shielded from the solvent, partially exposed to the solvent, or in the surface fully exposed to the solvent. From dimer to decamer, it was observed that increasing the oligomerization state results in a decrease in the SASA values of the Tyr residues, which indicates that overall, tyrosine residues are more shielded from the solvent when the oligomerization state increase. The SASA of the tyrosine residues in the dimer presents a bimodal distribution with peaks in ∼0.525 nm^2^ and ∼1.175 nm^2^, with a relative difference of 37 % (Figure 5A).

These values represent solvent‐exposed residues partially. The decamer presents a trimodal distribution of the SASA with the highest peak at ∼0.15 nm^2^, representing Tyr residues that are not exposed to the solvent. The other two lower peaks are ∼0.525 nm^2^ and ∼0.975 nm^2^, with a relative difference with the highest value of 48 % and 43 %, respectively. All the tyrosine residues are randomly distributed, and no individual preference was observed. We assessed the behavior of the Tyr residues of the 33‐mer peptide at the initial steps of self‐assembly through a characterization of the different rotamer species present in each trajectory.

In general, Tyr rotamers can populate three different rotamer states defined by the dihedral angles χ_1_ and χ_2_ formed by C_α_−C_β_ and C_β_−Cγ, respectively^[38]^ (Figure 5B), which are namely: the *g+* state with 0°<χ_1_120°, the *t* state with 120°< χ_1_<240° and the *g‐* state with 240°<χ_1_<360° and the second degree of freedom can be addressed by χ_2_ (Figure 5B).

The surrounding environment of each Tyr directly influenced the rotamer state, which has been proposed to produce distinct fluorescence lifetime contributions.[^31^, ^35b^, ^39^] The initial oligomers were analyzed by tracking the χ_1_and χ_2_ dihedral angles for all the Tyr residues in the last 100 ns of the simulations when the ensembles were stable. The significant change occurred in χ_1._ Therefore, this dihedral angle was empoleyed for the analysis.^[40]^ The Tyr residues on the dimer presented a preponderance of the *t* state with 49 % of the occurrences, closely followed by the *g‐* form with 46 % and the *g+* dihedral with only 7 % (Figure 5C). In the decamer, the χ_1_distribution of the 30 Tyr residues shows a shift towards *g ‐* states to the detriment of the *t* rotamer population with 53 % and 43 %, respectively. The *g*+ state remains poorly sampled at only 7 %. Our findings showed that the flexibility of 33‐mer oligomers, whose principal secondary structure is PPII, allows the Tyr rotamers to visit the energetically most favorable rotamers,^[38]^ in contrast to what was found for those Tyr residues in the α−helix of the Aβ‐amyloid oligomers, whose major contributor was the *g* + rotamer. ^[35,36]^ Regarding the χ_2_ dihedral, it predominantly visits, in both oligomers, the *g* state. This dihedral is degenerate for Tyr since changes of 180° represent the same rotamer, minor changes were observed.

With these results, we propose that the first stages of the oligomerization process are accompanied by a reduction in the SASA values of the Tyr residues, as well as an increase in the percentage of the *g‐* state and a decrease of the *t* state, as it is observed in the comparison between the dimer and the decamer. Although the MD simulations describe the process that can occur at the molecular level in a few molecules, the information obtained showed the possibility of using Tyr fluorescence decay measurements to monitor the transition from small oligomers to larger oligomers in the self‐assembly process of 33‐mer gliadin peptide.

## Conclusion

In this work, the self‐assembly of the 33‐mer peptide into thin‐plate structures was investigated using a combination of different techniques, including fluorescence spectroscopy, surface tension activity, microscopy, and MD calculations. We detected that the 33‐mer gliadin peptide is active at the water‐air interface, starting its self‐assembly above 75 nM. These results demonstrate that the 33‐mer peptide is a surfactant peptide. Studying the system in the micromolar range using intrinsic Tyr fluorescence as an intrinsic sensor, we detected the formation of 33‐mer peptide larger superstructures at 165 μM. The stabilized large thin‐plate structures coexist with spherical oligomers at 600 μM by Cryo‐TEM. As fluorescence spectroscopy of Tyr residues gives insight into the three‐dimensional environment of these residues, the results obtained here confirm the presence of 33‐mer nanoobjects and provide additional tools to monitor the formation of the 33‐mer thin plate structures. Finally, we described using computational approaches the possible changes that the Tyr residues in the 33‐mer gliadin peptide undergo during the self‐assembly process. By the simulation of two minimalist systems such as a dimer and decamer, we found that in the decamer, there is a change in the increment of the *t* state of Tyr towards *g‐*. These results might explain the changes observed in the lifetime distribution analysis of Tyr fluorescence decay times during the peptide self‐association process. Our findings may directly impact the biomedical evaluation of 33‐mer gliadin peptide in cellular models. The amphiphilic nature of the 33‐mer explains the controversial transport mechanism of the 33‐mer,^[21]^ showing that from a structural point of view, it is possible to obtain a fluid means of transport,^[5b]^ as well as transcellular or paracellular transport depending on the experimental conditions and peptide concentration.^[41]^ The nature of oligomers among the thin plates is not clear. Based on the recent discoveries in amyloid‐related diseases showing that the released oligomers from the fibrils are toxic species, it requires further investigation.[^29^, ^30^] Our current research efforts are directed toward understanding the behavior of thin‐plate structures and their oligomers in the cellular context, with the final aim to disclose their role in health and disease.

## Experimental Section

### Sample preparation

The 33‐mer gliadin peptide was synthesized, purified (95 % purity), and characterized as described before (Figure S1).[^7^, ^8^, ^10^] A mother stock solution of 33‐mer gliadin peptide was prepared at 766 μM in Milli‐Q water pH 6.5 and stabilized for at least 3 days at 4 °C. Then, the different solutions at the required concentrations were prepared by dilution, as reported before.[^7^, ^10^] Stabilized and freshly prepared samples were measured, but only the analysis from the stabilized samples was presented. As replicates, two stock solutions were prepared independently from two different peptide batches. Previously, it was found that the small intestine pH was in the range of 6.6 (0.5).^[42]^

### Steady‐state anisotropy

Samples of the 33‐mer peptide were analyzed using a fluorometer Fluoromax‐P (Glasgow, UK) from Horiba‐Jobin‐Yvon, coupled with polarizers. The samples were excited at 280 nm, and the fluorescence was collected at the different polarizer configurations at 305 nm at 25 °C, using 5 nm slits for the excitation and emission. For each configuration, the intensity values were obtained 10 times for each concentration, and anisotropy was calculated by the following equation Eq. 1.

(1) $$r=\;\left(I_{vv}-I_{vh}\right)/\left(I_{vv}+2GI_{vh}\right)$$

where r is the anisotropy, $I_{vv}$ is the intensity obtained where the polarizers are in the parallel, and $I_{vh}$ is the intensity obtained at the vertical.G is a correction factor which is calculated as: $\left(\;I_{vh}/I_{hh}\right)$ .

### Time‐correlated single‐photon counting

The decay curves of the tyrosine fluorescence of 33‐mer peptide at different concentrations were obtained using a fluorometer SLM‐AMINCO equipped with a pulsed diode laser PDL800‐B with an excitation wavelength of 282 nm. The pulse amplitude was 600 ps with a 5 MHz repetition. A filter at 283 nm was used after the sample to cut the excitation light, so only the emitted light was collected. The detection module used was from Pico Quant.

The fluorescence decays were obtained at the magic angle conditions, where the excitation polarizer was located at 0°, and the emission was at 54.7°. This was performed to avoid polarization effects. The impulse response function (IRF) was obtained by recording the decay of a glycogen solution to subtract the influence of the laser and detector on the measurement. The software Pico Harp 300 was employed for the data collection and analysis.

### Analysis of the fluorescence decay kinetics

**Non‐Linear Least square method (NLLS)**: The fluorescence decays obtained at the different peptide concentrations were analyzed using the Fluofit software from PicoQuant, which uses an exponential model to fit the data. The equation used for the analysis was Eq. 2:

(2) $$I\left(t\right)=\;\int_{-\infty }^{t}IRF\left(\;t'\right)\sum_{i=1}^{n}A_{i}e^{-\frac{\left(t-t'\right)}{\tau i}}d$$

where $I\left(t\right)$ is the intensity at time t, $IRF\left(\;t'\right)$ is the response function at the time t ' and $A_{i}$ is the pre‐exponential factor andτi is the lifetime. The goodness of the fitting was determined by the $\chi^{2}$ values, which were between 0.8 and 1.2.

**Maximum entropy method (MEM)**: The Maximum Entropy Method has been applied by using the MemExp Software available online.[^18a^, ^43^] In the Maximum Entropy Method, the experimental kinetics I(t) is fitted by the following function Eq. 3:

(3) $$I\left(t\right)=D_{0}\int_{-\infty }^{+\infty }[g(log\tau )]e^{-t/\tau }dlog\tau$$

where $g\left(\log\tau \right)$ is the lifetime distributions that correspond to decay kinetics. The fitting procedure entails the maximization of the function (Eq. 4)

Q=S-C-I

(4) Q=S-λC-αI

is the entropy defined as (Eq. 5)

(5) $$S\left(\vec{g},\vec{F}\right)=\sum_{j=1}^{M}\left[g_{j}-F_{j}-g_{j}ln\left(g_{j}/F_{j}\right)\right]\;$$

where *F* is the MEM prior distribution used to incorporate prior knowledge into the solution. *C* is a measure of the quality of the fit *F* to the data and corresponds to the χ^2^ dealing with normally distributed noise. I is a normalization factor; λ and α are Lagrange multipliers. The Maximum Entropy Method is a valuable tool in determining micro‐heterogeneity at the intra‐ and inter‐entities levels.[^37a^, ^44^]

The fuzzy entropy was obtained using the following equation (Eq. 6)[^37b^, ^45^]

(6) $$H=-\frac{1}{log\left(N\right)}\sum_{i=1}^{N}\left(w_{i}{}^{'}log\left(w_{i}{}^{'}\right)\right)$$

where $w_{i}{}^{'}$ represents the weight of the i‐th lifetime.

### Surface tension measurement

The critical aggregation concentration of 33‐mer gliadin peptide was determined using the Wilhelmy plate. Measurements were done at 22 °C with a DCAT 21 tensiometer from Dataphysics (Filderstadt, Germany) using a platinum‐iridium plate. After reaching buffer stability, titration experiments were carried out with additions of freshly filtered (220 nm cut‐off filter) 33‐mer gliadin peptide stock solutions using a micro‐syringe. A detailed description of the measurements is in the Supporting Information material.

### Cryo‐transmission electron microscopy

A 600 μM stabilized dispersion of the 33‐mer in Milli‐Q water pH 6.5 was deposited on lacey carbon filmed copper grids by blotting them with a filter paper. The resulting thin film was vitrified by quickly plunging the grids into liquid ethane at its freezing point using an automatic plunge freeze device (EM GP, Leica, Wetzlar, Germany). Specimens were examined at a temperature around 90 K with a Zeiss/LEO EM922 Omega TEM (Zeiss Microscopy, Oberkochen, Germany). Images were recorded by a CCD digital camera (Ultrascan 1000, Gatan, Garching, Germany) and analyzed using the GMS 1.8 software (Gatan). The size distribution was obtained using Image J.^[46]^

### Molecular dynamic simulations

The simulations were initialized with extended 33‐mer peptides constructed as reported previously.^[8]^ Each system was simulated for 250 ns in GROMACS 4.6.5 package.^[47]^ The force field employed was GROMOS53a6^[48]^ and the SPC/E model for the water.^[49]^ The simulations were performed with an explicit solvent and a concentration of 150 mM of Cl−Na+ ions. 3D periodic boundary conditions were imposed in cubic boxes, whose length dimensions were defined as the diameter of the peptide assembly plus 2.8 nm. The equations of motion were integrated with the leap‐frog algorithm at a 2‐fs time‐step, and the LINCS algorithm was used to constrain bonds to their correct lengths. The production simulations were performed in the isobaric‐isothermal (NPT) ensemble, using the Nose‐Hoover algorithm^[50]^ to keep the temperature at 310 (coupling constant t_T_ =0.8 ps). The Parrinello‐Rahman barostat^[51]^ to keep pressure isotropically constant at 1 bar (coupling constant t_T_ =2.0 ps and compressibility 4.5 ⋅ 10^5^ bar^−1^). A cut‐off scheme was employed for Lennard‐Jones interactions further than 1.2 nm. The particle mesh Ewald (PME) method was applied for electrostatic interactions with a real‐space cut‐off of 1.2 nm.

The protocol began with a minimization with the Steepest descent until the maximum force reached 200 kJ/mol nm, with an initial step of 0.01 nm. The minimization was followed by a two‐step equilibration with the position of the peptides restrained; first, a 0.2 ns simulation in the NVT ensemble was performed to relax the solvent molecules around the peptides and reach the desired simulation temperature while allowing the pressure to vary freely. The last equilibration step consisted of a 1 ns simulation of the system in the NPT ensemble. Finally, the production simulations of both systems were 250 ns.

The simulations were visualized with VMD and analyzed with GROMACS packages g_chi (gmx chi) to obtain the Tyrosine rotamers of the last 100 ns of the trajectories and g_sasa (gmx sasa) to compute the solvent‐accessible surface area of the Tyr residues in the same period. Figures were made with ggplot2^[52]^ implemented in R.^[53]^

## Conflict of interest

The authors declare no conflict of interest.

1

## Supporting information

As a service to our authors and readers, this journal provides supporting information supplied by the authors. Such materials are peer reviewed and may be re‐organized for online delivery, but are not copy‐edited or typeset. Technical support issues arising from supporting information (other than missing files) should be addressed to the authors.

Supporting Information

## Data Availability

The data that support the findings of this study are available from the corresponding author upon reasonable request.
