# Supplementary material for: Thin‐Plate Superstructures of the Immunogenic 33‐mer Gliadin Peptide
Source: Chembiochem. 2022 Oct 18;23(22):e202200552. doi: 10.1002/cbic.202200552 (PMC9828358; doi:10.1002/cbic.202200552)
Supplement: Supplementary file 1 — Supporting Information [file CBIC-23-0-s001.pdf]

ChemBioChem

Supporting Information

Thin-Plate Superstructures of the Immunogenic 33-mer Gliadin Peptide

Maria Georgina Herrera, Maria Julia Amundarain, Franscesco Nicoletti, Marcus Drechsler, Marcelo Costabel, Pier Luigi Gentili, and Veronica Isabel Dodero*

The 33-mer peptide

The 33-mer gliadin peptide LQLQPFPQPQLPYPQPQLPYPQPQLPYPQPQPF (3911 Da) with more than 95% purity was purchased lyophilised to Biochem Shanghai Ltd at different periods. Purity and mass were re-examined before experiments. Reverse-phase HPLC analysis was performed using a Venusil XBP C18 250 × 4.6. Binary gradients of solvents A (CH_3CN 0.1 %TFA) and B (H_2O 0.1%) were employed at a flow rate of 1.0 mL/min. The injection volume was 10 µL. HPLC peaks were detected by monitoring the UV absorbance at λ=220 nm, and the identity was confirmed by MS. The retention time of 10.240 min was obtained under the following experimental conditions: CH_3CN /H_2O with 0.1% TFA, 26-51%A 25 min. HPLC-MS (ESI): m/z 1305.16 $(M^+ + 3H)^{3+}$, 978.8 $(M^+ + 4H)^{4+}$. Accurate Mass determination has been performed by ESI nanoMS: Measured Ion Mass $(M^+ + 4H)^{4+}$: 978.26472 (deviation 0.10) and Calculated Ion Mass: 978.26463 (deviation 0.11); Molecular Formula Obtained $(C_{190}H_{273}N_{43}O_{47})\ H_4^{+4}$.

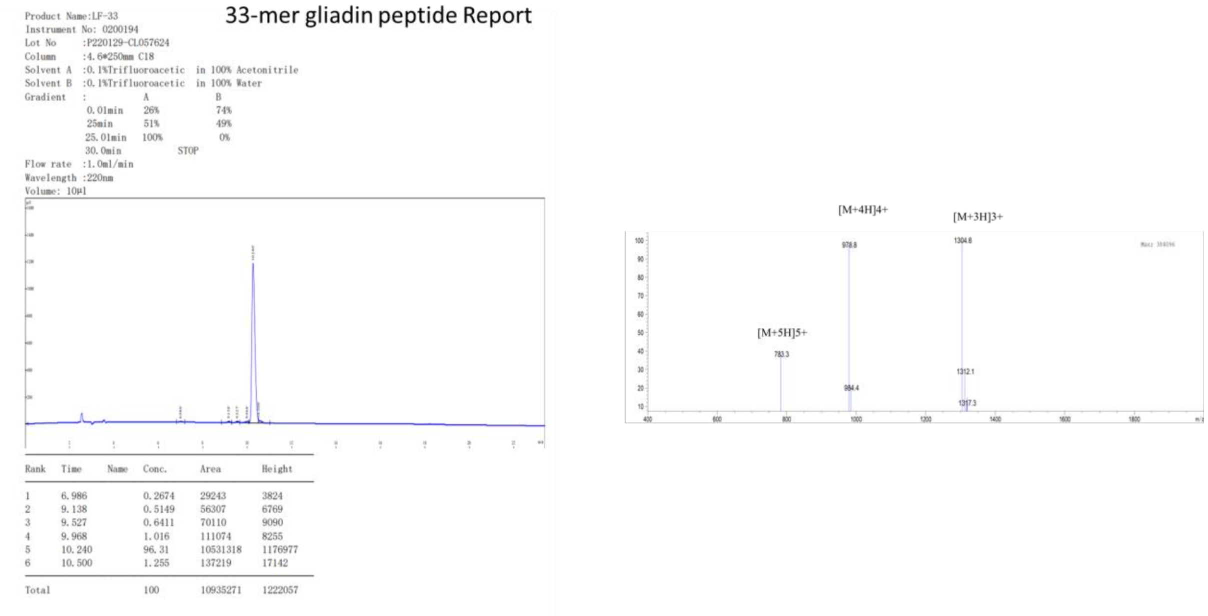

Rank	Time	Name	Conc.	Area	Height
1	6.986		0.2674	29243	3824
2	9.138		0.5149	56307	6769
3	9.527		0.6411	70110	9090
4	9.968		1.016	111074	8255
5	10.240		96.31	10531318	1176977
6	10.500		1.255	137219	17142
Total			100	10935271	1222057

Figure S1: The 33-mer gliadin peptide purity report.

Surface tension measurements

The surface tension experiments were carried out using Dataphysics DCAT21 Wilhelmy plate. A platinum-iridium rectangle shape was used as probe (length: 10 mm; width: 19.9 mm; thickness: 0.2 mm). The device was set with 1m/s speed and 3 mm immersion depth. All measurements were done at room temperature (RT), in a range of temperatures between 20°C and 22.5 C. Plastic Petri dishes

(diameter 3.5cm) were used as a bin for the samples. For the experiments, 5 ml of Milli-Q water, filtered with a 0.2 µm filter, was added stepwise to the Petri dish using a Gilson pipette. By diluting the 33-mer stock solution (766 µM) 1:10 in PBS, a freshly diluted 33-mer (76.6 µM) solution was prepared just prior to the experiments. The system was recorded until the plateau value of the surface tension was reached, followed by the subsequent addition of 33-mer aliquots using a micro-syringe (1-4 µl, with a maximum volume change of 3.2 %). Each measurement was calculated as the average of at least 50 recorded points and the instrument reading was set when the standard deviation was smaller than 0.03 mN/m. To obtain a homogeneous solution, every new point was measured after 5 minutes. The surface tension values are given as the average of three readings and were performed in duplicates.

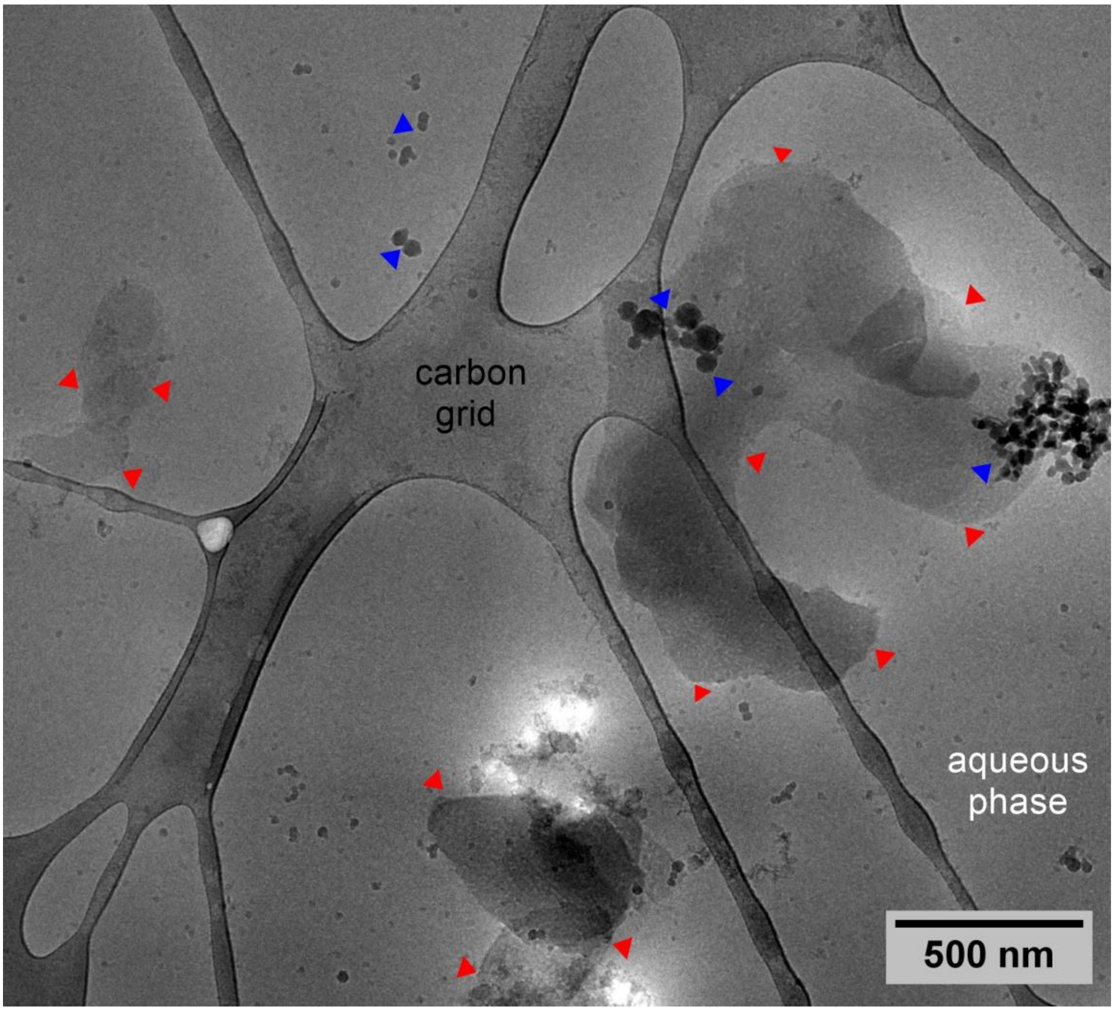

Figure S2: Low magnification Cryo-EM Image of the 33-mer thin plate-like structures (red arrows heads). Dark spots are ice contaminations (blue arrows head).

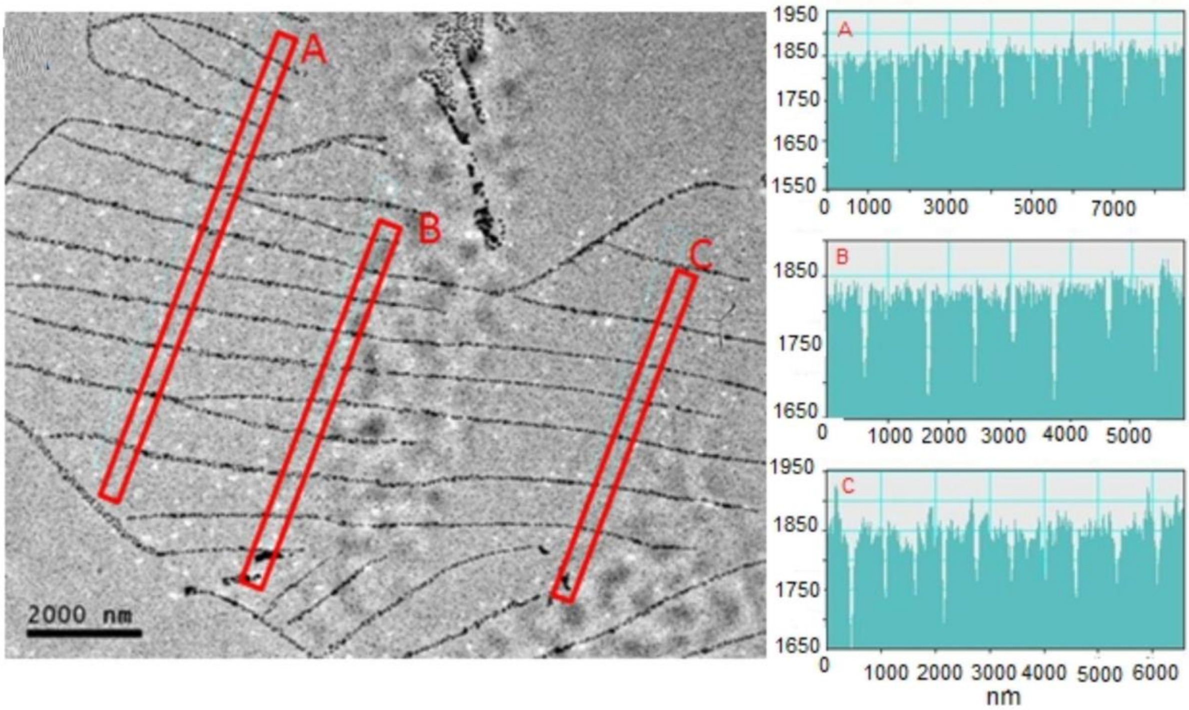

Figure S3: Low magnification TEM Image of the 33-mer peptide at 610 μM using cryo-fixation as reported before. The high contrast was achieved thanks to the transparency of the SiO2-coated grid. Panels A-C show the order of the linear aggregates in the whole field of view, in the background, it was detected isolated oligomers for more information see reference. (M. G. Herrera, M. Pizzuto, C. Lonez, K. Rott, A. Hutten, N. Sewald, J. M. Ruysschaert, V. I. Dodero, *Nanomedicine* **2018**, *14*, 1417-1427)
